# Four functional genotoxic marker genes (*Bax*, *Btg2*, *Ccng1*, and *Cdkn1a*) discriminate genotoxic hepatocarcinogens from non-genotoxic hepatocarcinogens and non-genotoxic non-hepatocarcinogens in rat public toxicogenomics data, Open TG-GATEs

**DOI:** 10.1186/s41021-024-00322-8

**Published:** 2024-12-19

**Authors:** Chie Furihata, Takayoshi Suzuki

**Affiliations:** 1 Division of Molecular Target and Gene Therapy Products, National Institute of Health Sciences, 3-25-26 Tonomachi, Kawasaki-Ku, Kawasaki, Kanagawa 210-9501 Japan; 2 School of Science and Engineering, Aoyama Gakuin University, Sagamihara, Sagamihara, Kanagawa 252-5258 Japan; 3 Division of Genome Safety Science, National Institute of Health Sciences, 3-25-26, Tonomachi, Kawasaki-Ku 210-9501 Japan

**Keywords:** Toxicogenomics, Genotoxic marker genes, Open TG-GATEs, Genotoxic carcinogen, Non-genotoxic carcinogen, Non-carcinogen

## Abstract

**Background:**

Previously, Japanese Environmental Mutagen and Genome Society/Mammalian Mutagenicity Study Group/Toxicogenomics Study Group (JEMS/MMS toxicogenomic study group) proposed 12 genotoxic marker genes (*Aen*, *Bax*, *Btg2*, *Ccnf*, *Ccng1*, *Cdkn1a*, *Gdf15*, *Lrp1*, *Mbd1*, *Phlda3*, *Plk2*, and *Tubb4b*) to discriminate genotoxic hepatocarcinogens (GTHCs) from non-genotoxic hepatocarcinogens (NGTHCs) and non-genotoxic non-hepatocarcinogens (NGTNHCs) in mouse and rat liver using qPCR and RNA-Seq and confirmed in public rat toxicogenomics data, Open TG-GATEs, by principal component analysis (PCA). On the other hand, the U.S. Environmental Protection Agency (US EPA) suggested seven genotoxic marker genes (*Bax*, *Btg2*, *Ccng1*, *Cgrrf1*, *Cdkn1a*, *Mgmt*, and *Tmem47*) with Open TG-GATEs data. Four genes (*Bax*, *Btg2*, *Ccng1*, and *Cdkn1a*) were common in these two studies. In the present study, we examined the performance of these four genes in Open TG-GATEs data using PCA.

**Results:**

The study's findings are of paramount significance, as these four genes proved to be highly effective in distinguishing five typical GTHCs (2-acetylaminofluorene, aflatoxin B1, 2-nitrofluorene, *N*-nitrosodiethylamine and *N*-nitrosomorpholine) from seven typical NGTHCs (clofibrate, ethanol, fenofibrate, gemfibrozil, hexachlorobenzene, phenobarbital, and WY-14643) and 11 NGTNHCs (allyl alcohol, aspirin, caffeine, chlorpheniramine, chlorpropamide, dexamethasone, diazepam, indomethacin, phenylbutazone, theophylline, and tolbutamide) by PCA at 24 h after a single administration with 100% accuracy. These four genes also effectively distinguished two typical GTHCs (2-acetylaminofluorene and *N*-nitrosodiethylamine) from seven NGTHCs and ten NGTNHCs by PCA on 29 days after 28 days-repeated administrations, with a similar or even better performance compared to the previous 12 genes. Furthermore, the study's analysis revealed that the three intermediate GTHC/NGTHCs (methapyrilene, monocrotaline, and thioacetamide, which were negative in the *Salmonella* test but positive in the in vivo rat liver test) were located in the intermediate region between typical GTHCs and typical NGTHCs by PCA.

**Conclusions:**

The present results unequivocally demonstrate the availability of four genotoxic marker genes ((*Bax*, *Btg2*, *Ccng1*, and *Cdkn1a*) and PCA in discriminating GTHCs from NGTHCs and NGTNHCs in Open TG-GATEs. These findings strongly support our recommendation that future rat liver in vivo toxicogenomics tests prioritize these four genotoxic marker genes, as they have proven to be highly effective in discriminating between different types of hepatocarcinogens.

**Supplementary Information:**

The online version contains supplementary material available at 10.1186/s41021-024-00322-8.

## Introduction

Carcinogenicity testing is important to identify carcinogens in environmental chemicals [1], chemicals in daily life [2], and pharmaceutical drug and pesticide development [3, 4]. However, evaluating carcinogenicity using conventional 2-year rodent-based animal studies is time-consuming and labor-intensive [5]. Thus, there is an increased need to develop novel alternative approaches to these rodent bioassays for assessing the carcinogenicity of substances [6].

Carcinogens have traditionally been classified into two categories according to their presumed mode of action: genotoxic (GTC) and non-genotoxic (NGTC). An OECD expert group defined GTCs as having the potential to induce cancer by interacting directly with DNA and the cellular apparatus involved in preserving the integrity of the genome. In contrast, NGTCs have the potential to induce cancer without interacting directly with either DNA or the apparatus mentioned above [7]. Genotoxic carcinogens are usually identified based on positive results in different in vitro and in vivo test systems. These include detecting DNA strand breaks (comet assay) [OECD TG489 (in vivo)], unscheduled DNA synthesis [OECD TG482 (in vitro), OECD TG486 (in vivo)], sister chromatid exchange [OECD TG479 (in vitro)], chromosomal aberrations [OECD TG473 (in vitro)], DNA adduct formation [8], mitotic recombination [9], and gene mutation [OECD TG476 (in vitro)]. Standard tests of mutagenicity include the Ames test [OECD TG471], in vitro metaphase chromosome aberration assay [OECD TG473], in vitro micronucleus assay [OECD TG487], L5178Y/tk ± mouse lymphoma (thymidine kinase) gene mutation assay [OECD TG490], in vivo micronucleus assay in rodents [OECD TG474], and transgenic rodent mutation assay [OECD TG488]. NGTCs show a threshold for exerting hazardous effects, and the various authoritative bodies set guidelines regarding appropriate exposure levels like other hazardous substances. They recommended clear differences between threshold and non-threshold carcinogens dealing with carcinogen classification and risk assessment [10].

Toxicogenomics, the application of transcription profiling to toxicology, has been widely used for elucidating the molecular and cellular actions of chemicals and other environmental stressors on biological systems, predicting toxicity before any functional damages, and classification of known or new toxicants based on signatures of gene expression. The success of a toxicogenomics study depends upon close collaboration among experts in different fields, including a toxicologist or biologist, a bioinformatician, a statistician, a physician, and, sometimes, a mathematician [11].

Previously, the JEMS/MMS toxicogenomics study group has been conducting studies using DNA microarray [12] and qPCR [13–17] to discriminate genotoxic hepatocarcinogens from non-genotoxic hepatocarcinogens in mice [13, 14, 16] and in rats [15] for use in an in vivo short-term toxicogenomics screening test for genotoxic carcinogens. We proposed 12 genotoxic marker genes (*Aen*, *Bax*, *Btg2*, *Ccnf*, *Ccng1*, *Cdkn1a*, *Gdf15*, *Lrp1*, *Mbd1*, *Phlda3*, *Plk2* and *Tubb4b*) to discriminate GTHCs from NGTHCs in mouse study using eight mouse GTHCs from five different functional groups [aromatic amines: 2-acetamidofluorene and 2,4-diaminotoluene; azobenzene: 4-dimethylaminoazobenzene; ester of carbamic acid: urethane; heterocyclic aromatic compound: quinoline; and nitrosamines: diisopropanolnitrosamine, 4-(methylnitrosamino)−1-(3-pyridyl) 1-butanone, and *N*-nitrosomorpholine] and four mouse non-genotoxic hepatocarcinogens [chlorinated aromatic hydrocarbons:1,4-dichlorobenzene and dichlorodiphenyltrichloroethane; phthalate: di(2-ethylhexyl) phthalate; and heterocyclic organic compound: furan] [14]. These seven mouse genotoxic hepatocarcinogens [18–24] except urethane [25] also induced hepatocellular carcinoma in rats. The results suggested that these carcinogens had similar modes of action in both rat and mouse liver. As described previously [14], nine (*Aen*, *Bax*, *Btg2*, *Ccng1*, *Cdkn1a*, *Gdf15*, *Mbd1*, *Phlda3*, and *Plk2*) of 12 marker genes are members of gene families related to the intrinsic apoptotic signaling pathway in response to DNA damage by the p53 class mediator. *Ccnf* may be related to DNA repair and DNA damage [26]. *Lrp1* may be related to tumor growth and metastasis, particularly by modulating three extracellular tumor environments [27]. *Tubb4b* may be related to metastasis in colon cancer [28]. Table 2 shows the symbol, gene name, and gene ID of the 12 corresponding rat genes. We confirmed the 12 genes in public rat toxicogenomics data, Open TG-GATEs [29]. We studied differentially expressed protein-coding genes with targeted RNA-Seq on freshly frozen rat liver tissues [30] and on formalin-fixed paraffin-embedded (FFPE) rat liver tissues [31, 32] after 28 days of treatment with chemicals using PCA.

The present study examined data from rat [male Crl:CD Sprague–Dawley (SD) rat, 6-week-old] Open TG-GATEs with newly selected genes. The Open Japanese Toxicogenomics Project-Genomics Assisted Toxicity Evaluation System (Open TG-GATEs) was developed by the Japanese Toxicogenomics Project (TGP) consortium and opened to the public in 2015 (http://toxico.nibiohn.go.jp/english/) [33]. The data include 170 chemicals. The data contain concurrent vehicle controls, three rats per group, three doses, and various time points (3, 6, 9, and 24 h after a single administration and 4, 8,15, and 29 days after repeated administrations).

The registered data include five typical GTHCs [2- acetamidofluorene (AAF), aflatoxin B1 (AFL), 2-nitrofluorene (2NF), *N*-nitrosodiethylamine (DEN) and *N*-nitrosomorpholine (NNM)] and seven typical NGTHCs [clofibrate (CLO), ethanol (ETH), fenofibrate (FEN), gemfibrozil (GEM), hexachlorobenzene (HEX), phenobarbital (PHE) and WY-14643 (WY)] for comparison. Among them, three genotoxic hepatocarcinogens (AFL: mycotoxin; 2NF: nitrated polycyclic aromatic hydrocarbon; and NNM: nitrosamine) and all seven NGTHCs are different from those in our previous study [14]. Seven NGTHCs were fibric acid derivatives: CLO and GEM; simple alcohol: ETH; synthetic phenoxy-isobutyric acid derivate: FEN; chlorinated aromatic hydrocarbon: HEX; barbituric acid derivative: PHE; and thioacetic acid derivative: WY, a peroxisome proliferator. Six NGTHCs, except HEX, have functional groups different from those of our four previous NGTHCs.

The IWGT-Toxicogenomics meeting was held at ICEM in Toronto in 2022, where 12 genotoxicity marker genes from JEMS/MMS and seven marker genes from EPA [34] were discussed. Four genes (*Bax*, *Btg2*, *Ccng1*, and *Cdkn1a*) were common in these two studies. Then, we analyzed the four genes in the rat public toxicogenomics data “Open TG-GATEs”.

We analyzed 23 previous chemicals (five GTHCs, seven NGTHCs, and eleven NGTNHCs) [29] and three intermediate GTHCs/NGTHCs. We designated that typical GTHCs are mutagenic in the *Salmonella test* and genotoxic in some in vivo liver tests, such as the micronucleus test, the transgenic rodent mutation assay, and the UDS test and carcinogenic in rat liver. Typical NGTHCs are not mutagenic in the *Salmonella* test, do not show genotoxicity in in vivo rat liver tests, and are carcinogenic in rat liver. NGTNHCs are not mutagenic in the *Salmonella* test and are not carcinogenic in rat liver. Intermediate GTHC/NGTHCs are negative in the *Salmonella test* and show contradictory results with in vivo rat liver tests. Table 1 summarizes this study's classification of GTHC, NGTHC, NGTNHC, and intermediate GTHC/NGTHC [35–77].

**Table 1:** Examined GTHCs, NGTHCs, NGTNHCs and intermediate GTHCs/NGTHCs in the present study

No	Classification	Chemical	Abbreviations	*Salmonella test*	Male rat in vivo liver test	Carcinogenicity in malle rat liver	Carcinogenicity in male rat in other organs	IARC group ^35^	References
1	**GTHCs**	2-acetylaminofluorene	AAF	+	MN + , UDS +	+	mgl, ski	NL	36
2		aflatoxin B1	AFB	+	UDS +	+	-	1	36, 37, 38
3		2-nitrofluorene	2NF	+	UDS +	+	kid, sto	2B	36, 39
4		*N*-nitrosodiethylamine	DEN	+	UDS + , DNA single strand scission +	+	eso, kid, vsc	2A	36, 40, 41
5		*N*-nitrosomorpholine	NNM	+	UDS +	+	kid, adr, thy, pit	2B	36, 41–43
6	**NGTHCs**	clofibrate	CLO	-	Pig-a -, UDS -, DSB -, MN +	+	pan, smi, dermatofibrosarcoma, tes	3	36, 44–47
7		ethanol	ETH	-	comet -, DNA strand break +	+	adr, pan, pit	1	36, 48, 49
8		fenofibrate	FEN	-	comet + (weak), ND (UDS, MN)	+	pan, tes	NL	50, 51
9		gemfibrozil	GEM	-	ND	+	adr, pan, tes	3	36, 52, 53
10		hexachlorobenzene	HEX	-	(Dominant lethal test -)	+	bile duct, kid	2B	36, 54
11		phenobarbital	PHE	-	MN -, comet -, Pig-a -	+	adr, nas	2B	36, 45, 55, 56
12		WY-14643	WY	-	UDS -	+	Leydig cell, pancreatic acinar cell	NL	57, 58
13	**NGTNHCs**	allyl alcohol	AA	-	ND	-	-	NL	36, 59
14		aspirin	ASP	-	ND	-	-	NL	36, 60
15		caffeine	CAF	-	ND	-	-	3	36, 61
16		chlorpheniramine	CPA	-	ND	-	-	NL	36, 62
17		chlorpropamide	CPP	-	ND	-	-	NL	36, 63
18		dexamethasone	DEX	-	ND	-	-	NL	36, 64
19		diazepam	DIA	-	ND	-	-	3	36, 65
20		indomethacin	IND	-	ND	-	-	NL	36, 66
21		phenylbutazone	PBZ	-	ND	-	-	3	36, 67
22		theophylline	THE	-	ND	-	-	3	36, 68, 69
23		tolbutamide	TOL	-	ND	-	-	NL	36, 70
24	**intermediate**	methapyrilene	MP	-	MN + , comet -, UDS -	+	-	NL	36, 55, 71, 72
25	**(GTHCs/NGTHCs)**	monocrotaline	MCT	-	MN + , DNA-DNA interstrand crosslinks + ,	+	-	2B	36, 73, 74
26		thioacetamide	TAA	-	comet + , γ-H2AX + , MN -	+	bile duct	2B	36, 75–77

*DSB *DNA strand breaks, *ND *Not determied by UDS, MN and comet assays, *NL *Not listed

*adr *adrenal gland, *eso *esophagus, *kid *kidney, *mgl *mammary gland, *nas *nasal cavity, pan: pancreas, *pit *pituitary gland, *ski* skin, *smi *small , *sto *stomach, *tes *testes, *thy *thyroid gland, *vsc *vascular system

In the present study, we propose that the four genes (*Bax*, *Btg2*, *Ccng1*, and *Cdkn1a*) instead of the previous twelve genes (*Aen*, *Bax*, *Btg2*, *Ccnf*, *Ccng1*, *Cdkn1a*, *Gdf15*, *Lrp1*, *Mbd1*, *Phlda3*, *Plk2*, and *Tubb4b*) are functional to discriminate genotoxic hepatocarcinogens from non-genotoxic hepatocarcinogens and non-genotoxic non-hepatocarcinogens in rat liver. The present study strongly supports our recommendation that future rat liver in vivo toxicogenomics tests prioritize these four genotoxic marker genes, as they have proven highly effective in discriminating between different types of hepatocarcinogens.

## Methods

### Chemicals

We analyzed data of the 26 chemicals: five typical GTHCs, seven typical NGTHCs, 11 NGTNHCs [29], and three intermediate GTHC/NGTHCs from Open TG-GATEs. Table 1 summarizes this study's classification of GTHC, NGTHC, NGTNHC, and intermediate GTHC/NGTHC [35–77]. Open TG-GATEs presented five typical GTHCs: 2-acetamodofluorene (CAS 53–96-3, AAF), aflatoxin B1 (CAS 1402–68-2, AFL, IARC Group 1), 2-nitrofluorene (CAS 607- 57–8, 2NF, IARC Group 2B), *N*-nitrosodiethylamine (CAS 55–18-5, DEN, IARC Group 2 A), and *N*-nitrosomorpholine (CAS 59–89-2, NNM, IARC Group 2B) at 24 h after a single administration and two typical GTHCs: AAF and DEN at 29 days after repeated administration. Open TG-GATEs presented seven typical NGTHCs after at 24 h after a single administration and at 29 days after repeated administration: four PPARα agonists [clofibrate (CAS 637–07-0, CLO, IARC Group 3), fenofibrate (CAS 49562–28-9, FEN), gemfibrozil (CAS 25812- 30–0, GEM, IARC Group 3), and WY-14643 (CAS 50892–23-4, WY)], two enzyme inducers [hexachlorobenzene (CAS 118–74-1, HEX, IARC Group 2B) and phenobarbital (CAS 50–06-6, PHE, IARC Group 2B)] and ethanol (CAS 64–17- 5, ETH, IARC Group 1), which induced oxidative stress. Open TG-GATEs presented the data of many NGTNHCs, but we selected 11 familiar chemicals in this study. NGTNHCs do not include carcinogens to other organs than liver [36].They are allyl alcohol (CAS 107–18-6, AA), aspirin (CAS 50–78-2, ASP), caffeine (CAS 58–08-2, CAF, IARC Group3), chlorpheniramine (CAS 113–92-8, CPA), chlorpropamide (CAS 94–20-2, CPP), dexamethasone (CAS 50–02-2, DEX), diazepam (CAS 439–14-5, DIA, IARC Group 3), indomethacin (CAS 53–86-1, IND), phenylbutazone (CAS 50–33-9, PBZ, IARC Group 3), theophylline (CAS 58–55-9, THE, IARC Group 3), and tolbutamide (CAS 64–77-7, TOL) at 24 h after a single administration. Open TG-GATEs examined 10 of the 11 NGTNHCs except DEX 29 days after repeated administration. Open TG-GATEs presented three intermediate GTHC/NGTHCs: methapyrilene (CAS 91–80-5, MP), monocrotaline (CAS 315–22-0, MCT, IARC Group 2B), and thioacetamide (CAS 62–55-5, TAA, IARC Group 2B) at 24 h after a single administration and at 29 days after repeated administration. They are negative in the *Salmonella* test [36] but positive in the in vivo rat liver test [71–77]. AAF was a metabolite of 2NF [78]. The IARC classification does not contain FEN, WY, AA, ASP, CPA, CPP, DEX, IND, MP, and TOL.

### Analyzed genes

Table 2 shows the symbol, gene name, and NCBI gene ID of the 12 analyzed rat genes.

**Table 2:** Twelve genes analyzed in the present study

No	Symbol	Gene name	Gene ID
1	Aen	apoptosis enhancing nuclease	361,594
2	Bax	BCL2 associated X, apoptosis regulator	24,887
3	Btg2	BTG anti-proliferation factor 2	29,619
4	Ccnf	cyclin F	117,524
5	Ccng1	cyclin G1	25,405
6	Cdkn1a	cyclin-dependent kinase inhibitor 1A	114,851
7	Gdf15	growth differentiation factor 15	29,455
8	Lrp1	LDL receptor related protein 1	299,858
9	Mbd1	methyl-CpG binding domain protein 1	291,439
10	Phlda3	pleckstrin homology-like domain, family A, member 3	363,989
11	Plk2	polo-like kinase 2	83,722
12	Tubb4b	tubulin, beta 4B class IVb	296,554

### Data analysis

We obtained relative fluorescence intensities of DNA microarray results of three individual rats in each dose- and time-group on the four genes from Open TG-GATEs　(http://toxico.nibiohn.go.jp/english/). Data from Open TG-GATEs were transferred into log2 data using the “R Project for Statistical Computing” (https://www.r-project.org/) to stabilize the variance. Ratio (exp/cont) log2 was calculated against the mean from the control group (Appendix A.1, A.2, B1, B2, C1, and C2). Discrimination of GTHCs vs. NGTHCs plus NGTNHCs was achieved by statistical analysis using the PCA program from the “R Project for Statistical Computing” (Appendix A.3, A.4, B3, B4, C3, and C4) as described previously [29]. Data for each dose in supplements and each point in figures presented in this paper are averages for three rats.

## Results

### Four genotoxic marker genes discriminate typical GTHCs from typical NGTHCs and NGTNHCs at 24 h after a single administration and 29 days after repeated administrations

**Fig.1:**
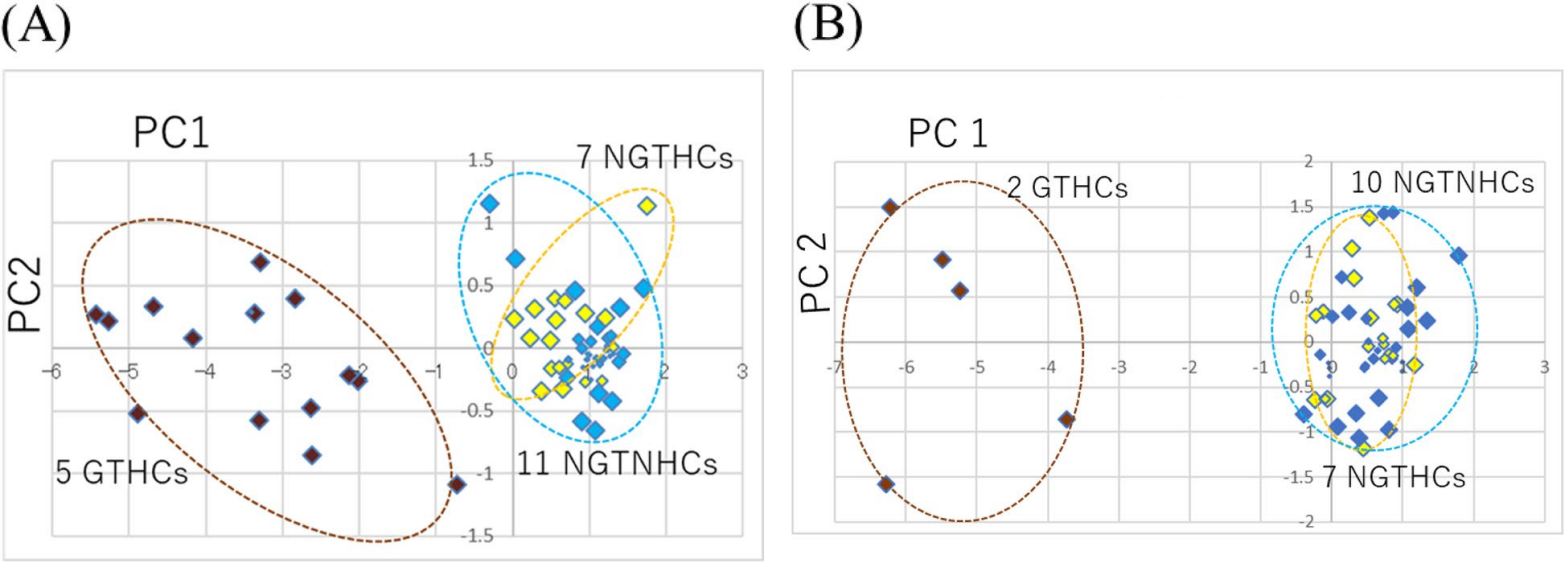
**A** and **B** Analysis of rat liver toxicogenomics public data (OPEN TG-GATEs, DNA microarray) by PCA with the four genes. Discrimination of GTHCs from NGTHCs and NGTNHCs at 24 h after a single administration [Fig. 1 (A)] and 29 days after repeated administrations [Fig. 2 (B)] by PCA with the four genotoxic marker genes (*Bax*, *Btg2*, *Ccng1*, and *Cdkn1*). The gene expression profile (log2) was calculated: the mean of each control group was calculated as 0 (log2), and the ratio (exp/cont) (log2) was calculated (Appendix A.1 for 24 h and Appendix A.3 for 29 days). These numerical values were analyzed by PCA (Appendix A.2 for 24 h and Appendix A.4 for 29 days). At 24 h, Fig. 1(A) shows the results of 23 chemicals with three doses, a total of 69 points: five typical GTHCs (brown-colored, AAF, AFL, DEN, NNM, and 2NF) were discriminated from seven typical NGTHCs (yellow-colored, CLO, ETH, FEN, GEM, HEX, PHE, and WY) and 11 NGTNHCs (blue-colored, AA, ASP, CAF, CPA, CPP, DEX, DIA, IND, PBZ, THE, and TOL). At 29 days, Fig. 1(B) shows the results of 19 chemical, total of 55 points: two typical GTHCs (brown-colored, AAF-low, -middle, and -high; DEN-low and -) were discriminated from seven typical NGTHCs (yellow-colored, CLO, ETH, FEN, GEM, HEX, PHE, and WY with three doses) and 10 NGTNHCs (blue-colored, AA, ASP, CAF, CPA, CPP, DIA, PBZ, THE, and TOL with three doses and IND-low and -middle). Each group is enclosed with an optional dashed ellipse. Five GTHCs [AAF: 2- acetamidofluorene, AFL: aflatoxin B1, 2NF: 2-nitrofluorene, DEN: *N*-nitrosodiethylamine and NNM: N-nitrosomorpholine], seven NGTHCs [CLO: clofibrate, ETH: ethanol, FEN: fenofibrate, GEM: gemfibrozil, HEX: hexa-chlorobenzene, PHE: phenobarbital, and WY: WY-14643], and 11 NGTNHCs (mostly pharmaceutical drugs) [AA: allyl alcohol, ASP: aspirin, CAF: caffeine, CPA: chlorpheniramine, CPP: chlorpropamide, DEX: dexamethasone, DIA: diazepam, IND: indomethacin, PBZ: phenylbutazone, THE: theophylline, and TOL: tolbutamide]

At 24 h after a single administration, as Open TG-GATEs presented five typical GTHCs (AAF, AFL, DEN, 2NF, and NNM) and seven typical NGTHCs (CLO, ETH, FEN, GEM, HEX, PHE, and WY), we analyzed five typical GTHCs, seven typical NGTHCs, and optional 11 NGTNHCs (AA, ASP, CAF, CPA, CPP, DEX, DIA, IND, PBZ, THE, and TOL), with three doses, 23 chemicals, 69 data points. Appendix A.1 shows the gene expression profile (log2), Appendix A.2 shows the first principal component (PC1) and the second principal component (PC2) analyzed by PCA, and Fig. 1(A) shows the result of PCA of five typical GTHCs, seven typical NGTHCs, and 11 NGTNHCs 24 h after a single administration in a two-dimensional figure. Five typical GTHCs are separated from seven typical NGTHCs and 11 NGTNHCs by PCA in a two-dimensional graph, with (PC1), where GTHCs exhibit PC1 below –0.719 (DEN-low), and NGTHCs exhibit PC1 above −0.292 (DIA-high) (Appendix A.2). PC1 of DEN was –0.719, −2.611, −4.885 for DEN-low, DEN-middle and DEN-high respectively, and reflected dose-dependent changes in gene expressions. The distinction between GTHCs and NGTHCs by the four genes is not inferior to, but somewhat superior to, the distinction by the 12 genes presented previously [reference 29, Fig. 4(C)].

At 29 days after repeated administration, as Open TG-GATEs presented two typical GTHCs and seven typical NGTHCs, we analyzed two typical GTHCs (AAF-low, AAF-middle, AAF-high, DEN-low, and DEN-middle), seven typical NGTHCs with three doses, and optional choice of the 10 NGTNHCs (AA, ASP, CAF, CPA, CPP, DIA, PBZ, THE, TOL with three doses, and IND-low and IND-middle), 19 chemicals, 55 data points. Appendix A.3 shows the gene expression profile (log2), Appendix A.4 shows the first principal component (PC1) and the second principal component (PC2) analyzed by PCA, and Fig. 1(B) presents the result of PCA on two typical GTHCs, DEN and AAF, seven typical NGTHCs, and 10 NGTNHCs. Figure 1(B) demonstrates the discrimination of two typical GTHCs from seven typical NGTHCs and 10 NGTNHCs with PC1 by PCA in a two-dimensional graph, where GTHCs exhibit PC1 below −3.724 (DEN-low), and NGTHCs and NGTNHCs exhibit PC1 above −0.388 (CPA-high) (Appendix A.4). The distinction of GTHCs from NGTHCs plus NGTNHCs by four genes is no different from the distinction by 12 genes presented previously [reference 29, Fig. 4(F)].

Our research has uncovered a novel aspect of genotoxicity. We have identified four genotoxic marker genes that discriminate GTHCs from NGTHCs and NGTNHCs. This distinction is observed 24 h after a single administration and 29 days after repeated administration, significantly advancing our understanding of genetic mechanisms.

### Three intermediate GTHC/NGTHCs are located in the intermediate region between typical GTHCs and typical NGTHCs by PCA with the four genes

In the current study, three additional intermediate GTHC/NGTHCs, methapyrilene (MP), monocrotaline (MCT), and thioacetamide (TAA), are analyzed. They are negative in the *Salmonella* test but positive in the in vivo rat liver test [32–34].

**Fig. 2:**
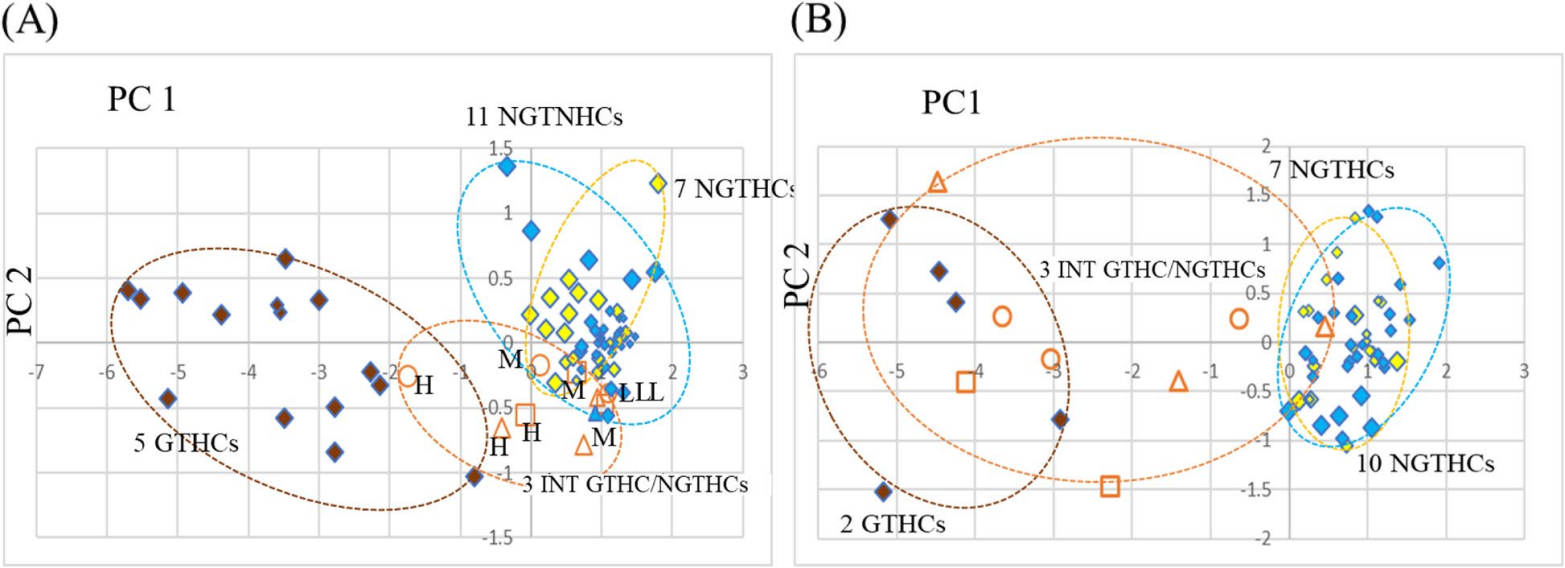
**A** and **B** Analysis of three intermediate GTHC/NGTHCs in rat liver toxicogenomics public data (OPEN TG-GATEs) by PCA with the four genes. Three additional intermediate GTHC/NGTHCs, methapyrilene (MP), monocrotaline (MCT), and thioacetamide (TAA), were analyzed with typical GTHCs, typical NGTHCs and NGTNHCs at 24 h after a single administration [Fig. 2(A)] and at 29 days after repeated administration [Fig. 2(B)] by PCA with four genotoxic marker genes (*Bax*, *Btg2*, *Ccng1*, and *Cdkn1*). The gene expression profile (log2) was calculated: the mean of each control group was calculated as 0 (log2), and ratio (exp/cont) (log2) was calculated (Appendix B.1 for 24 h and Appendix B.3 for 29 days). These numerical values were analyzed by PCA (Appendix B.2 for 24 h and Appendix B.4 for 29 days). At 24 h, Fig. 2(A) shows the results of 26 chemicals with three doses (low, middle and high), total of 78 points: five typical GTHCs (brown-colored, AAF, AFL, DEN, NNM, and 2NF) were discriminated from seven typical NGTHCs (yellow-colored, CLO, ETH, FEN, GEM, HEX, PHE, and WY) and 11 NGTNHCs (blue-colored, AA, ASP, CAF, CPA, CPP, DEX, DIA, IND, PBZ, THE, and TOL). GTHCs exhibit PC1 below –0.789 (DEN-low; PC1: −0.789, PC2: −1.035). NGTHCs and NGTNHCs exhibit PC1 above −0.334 (IND-high; PC1: −0.334, PC2: 1.355) (Appendix B.2). Speaking of the three intermediates GTHC/NGTHCs (MCT: □, MP: △, and TAA: 〇), TAA-high (PC1: −1.742, PC2: −0.251) is in the GTHCs area (below PC1: −0.789). MP-high (PC1: −0.408, PC2: −0.659), MCT-high (PC1: −0.075, PC2: −0.557) and MP-middle (PC1: 0.747, PC2: –0.79) are in the intermediate area (orange dashed circle). TAA-middle (PCA: 0.135, PC2: −0.173), TAA-low (PC1: 1.101, PC2: −0.385), MP-low (PC1: 0.952, PC2: −0.424), MCT-middle (PC1: 0.851, PC2: −0.231), MCT-high (PC1: 1.043, PC2: −0.47) and MCT-middle (PC1: 0.851, PC2: −0.231) are in the NGTHCs and NGTNHCs area. At 29 days, Fig. 2(B) shows the results of 22 chemical, total of 63 points: two typical GTHCs (brown-colored, AAF-low, -middle, and -high; DEN-low and -high) were discriminated from seven typical NGTHCs (yellow-colored, CLO, ETH, FEN, GEM, HEX, PHE, and WY with three doses) and 10 NGTNHCs (blue-colored, AA, ASP, CAF, CPA, CPP, DIA, PBZ, THE, and TOL with three doses and IND-low and -middle). GTHCs exhibit PC1 below −2.906 (DEN-low). NGTHCs and NGTNHCs exhibit PC1 above 0.001 (CPA-high) (Appendix B.4). Three intermediate GTHC/NGTHCs, MCT-middle (PC1: −4.125, PC2: −0.409), MP-high (PC1: −4.486, PC2: 1.642), TAA-middle (PC1: −3.043, PC2: −0.158), and TAA-high (PC1: −3.667, PC2: 0.274), are in the GTHCs area. In contrast, MCT-low (PC1: −2.279, PC2: −1.467), MP-middle (PC1: −1.403, PC2: −0.398) and TAA-low (PC1: −0.651, PC2: 0.248) are in the intermediate area between GTHCs and NGTHC and NGTNHCs. Only MP-low (PC1: 0.454, PC2: 0.163) is in the NGTHCs and NGTNHCs area. Each group is enclosed with an optional dashed ellipse. Five GTHCs [AAF: 2- acetamidofluorene, AFL: aflatoxin B1, 2NF: 2-nitrofluorene, DEN: *N*-nitrosodiethylamine and NNM: N-nitrosomorpholine], seven NGTHCs [CLO: clofibrate, ETH: ethanol, FEN: fenofibrate, GEM: gemfibrozil, HEX: hexa-chlorobenzene, PHE: phenobarbital, and WY: WY-14643], 11 NGTNHCs (mostly pharmaceutical drugs) [AA: allyl alcohol, ASP: aspirin, CAF: caffeine, CPA: chlorpheniramine, CPP: chlorpropamide, DEX: dexamethasone, DIA: diazepam, IND: indomethacin, PBZ: phenylbutazone, THE: theophylline, and TOL: tolbutamide] and three intermediate GTHC/NGTHCs [MCT: monocrotaline, MP: methapyrilene, and TAA: thioacetamide]

At 24 h after a single administration of Open TG-GATEs data, we analyzed five typical GTHCs (AAF, AFL, DEN, 2NF, and NNM), seven typical NGTHCs, optional choice of the 11 NGTNHCs (AA, ASP, CAF, CPA, CPP, DEX, DIA, IND, PBZ, THE, and TOL), and three intermediate GTHC/NGTHCs (MCT, MP, and TAA), with three doses, 26 chemicals, 78 data points. Appendix B.1 shows the gene expression profile (log2) of the four genes, Appendix B.2 shows the first principal component (PC1) and the second principal component (PC2) analyzed by PCA, and Fig. 2 (A) presents the result of PCA on five typical GTHCs, seven typical NGTHCs, 11 NGTNHCs, and three intermediate GTHC/NGTHCs 24 h after a single administration. The PCA results separate five typical GTHCs from seven typical NGTHCs and 11 NGTNHCs by PCA with PC1 in a two-dimensional graph, where GTHCs exhibit PC1 below –0.789 (DEN-low; PC1: −0.789, PC2: −1.035). NGTHCs and NGTNHCs exhibit PC1 above −0.334 (IND-high; PC1: −0.334, PC2: 1.355) (Appendix B.2). Speaking of the three intermediates GTHC/NGTHCs, TAA-high (PC1: −1.74, PC2: −0.251) is in the GTHCs area, and MCT-high (PC1: −0.075, PC2: −0.557), MP-high (PC1: −0.408, PC2: −0.659), and MP-middle (PC1: 0.747, PC2: −0.79) are in the intermediate area. TAA-low (PC1: 1.10, PC2: −0.385), TAA-middle (PC1: 0.135, PC2: −0.173), MP-low (PC1: 0.952, PC2: −0.424), MCT-middle (PC1: 0.651, PC2: −0.231), and MCT-low (PC1: 1.043, PC2: −0.47) are in the NGTHCs and NGTNHCs area. The results show that the three intermediate GTHC/NGTHCs、MCT, MP, and TAA span the GTHCs, intermediate, and NGTHCs and NGTNHCs areas at 24 h after a single administration.

At 29 days, Open TG-GATEs presented only two GTHCs. We analyzed two typical GTHCs (AAF with three doses and DEN-low and DEN-middle), seven typical NGTHCs with three doses, optional 10 NGTNHCs (AA, ASP, CAF, CPA, CPP, DIA, PBZ, THE, TOL with three doses, and IND-low and IND-middle), and three intermediate GTHC/NGTHCs (MCT-low, MCT-middle, MP-low, MP-middle, MPP-high, TAA-lor, TAA-middle, and TAA-high), 22 chemicals, 63 points. Appendix B.3 presents the gene expression profile (log2), Appendix B.4 presents a first principal component (PC1) and a second principal component (PC2) analyzed by PCA, and Fig. 2(B) presents the result of PCA on two typical GTHCs, seven typical NGTHCs, 10 NGTNHCs, and three intermediate GTHC/NGTHCs 29 days after repeated administration. Figure 2(B) shows the discrimination of two GTHCs from seven NGTHCs and 10 NGTNHCs with PC1 by PCA in a two-dimensional graph, where GTHCs exhibit PC1 below −2.906 (DEN-low). NGTHCs and NGTNHCs exhibit PC1 above 0.001 (CPA-high) (Appendix B.4). Speaking of the three intermediate GTHC/NGTHCs, MCT-middle (PC1: −4.125, PC2: −0.409), MP-high (PC1: −4.486, PC2: 1.642), TAA-middle (PC1: −3.043, PC2: −0.158), and TAA-high (PC1: −3.667, PC2: 0.274) are in the GTHCs area. In contrast, MCT-low (PC1: −2.279, PC2: −1.467), MP-middle (PC1: −1.403, PC2: −0.398) and TAA-low (PC1: −0.651, PC2: 0.248) are in the intermediate area between GTHCs and NGTHC and NGTNHCs. Only MP-low (PC1: 0.454, PC2: 0.163) is in the NGTHCs and NGTNHCs area. The results show that the three intermediate GTHC/NGTHCs、MCT, MP, and TAA, span the GTHCs and intermediate areas except MP-low after repeated administrations for 28 days. The 29-day results show that the three intermediate GTHC/NGTHCs are moving in the GTHCs and intermediate regions compared to the 24-h results.

### Comparison of analysis of three intermediate GTHC/NGTHCs analyzed by PCA with the 12 previously presented genes

In the previous paper [29], we presented an analysis of a total of 23 chemicals, 69 data points, five typical GTHCs, seven typical NGTHCs, and 11 optional NGTNHCs at 24 h after a single administration, and a total of 19 chemicals, 55 data points, except for three intermediate GHCs/NGTHCs. Therefore, we only present the result of 23 chemicals plus three intermediate GHCs/NGTHCs, a total of 26 chemicals, 78 data points, analyzed with 12 previously presented genes at 24 h after a single administration, and a total of 22 chemicals, 63 data points, 29 days after repeated administrations, in the present paper.

**Fig. 3:**
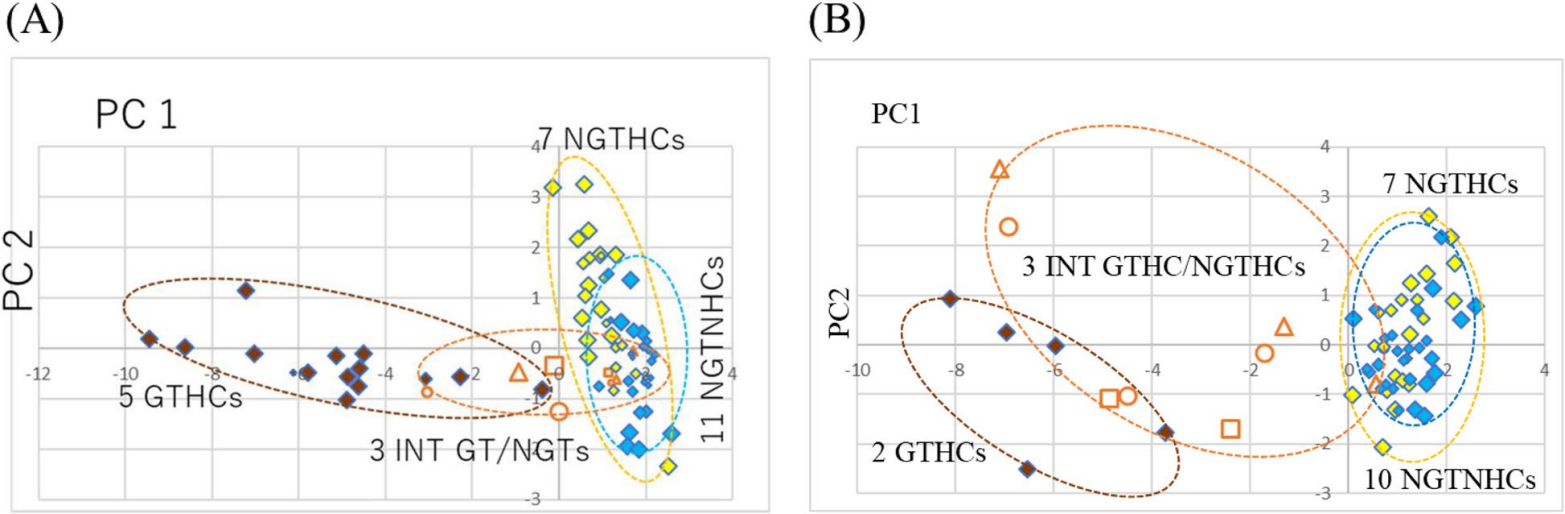
**A** and **B** Comparison of three intermediate GTHC/NGTHCs analyzed by PCA with the 12 previously presented genes. We analyzed 23 chemicals plus three intermediate GHCs/NGTHCs, a total of 26 chemicals, 78 points, analyzed by PCA with 12 previously presented genes [29] at 24 h after a single administration and a total of 22 chemicals, 63 points, at 29 days after repeated administrations. The gene expression profile (log2) was calculated: the mean of each control group was calculated as 0 (log2), and ratio (exp/cont) (log2) was calculated (Appendix C.1 for 24 h and Appendix C.3 for 29 days). These numerical values were analyzed by PCA (Appendix C.2 for 24 h and Appendix C.4 for 29 days). At 24 h after a single administration, The PCA result shows a clear separation of five typical GTHCs from seven typical NGTHCs and 11 NGTNHCs by PCA with PC1, where GTHCs exhibit PC1 below –0.381 (DEN-low, PC1: −0.381, PC2: −0.822), and NGTHCs and NGTHCs exhibit PC1 above −0.130 (NGTHC, FEN-high, PC1: −0.13, PC2: 3.184) (Appendix C.2). Speaking of the three intermediate GTHC/NGTHCs (MCT: □, MP: △, and TAA: 〇), MP-high (PC1: −0.941, PC2: −0.472) and TAA-high (PC1: −3.04, PC2: −0.869) are in the GTHCs area. MCT-high (PC1: −0.109, PC2: −0.353) and TAA-middle (PC1: −0.024, PC2: −1.236) are in the intermediate area. MCT-low (PCA: 1.688, PC2: −0.149), MCT-middle (PC1: 1.149, PC2: −0.489), MP-low (PC1: 1.702, PC2: −0.059), MP-middle (PC1:1.324, PC2: −0.618), and TAA-low (PC1: 1.205, PC2: −0.671) are in the NGTHCs and NGTNHCs area. The analysis with12 genotoxic marker genes are similar to that with four genotoxic marker genes [Fig. 2(A)]. At 29 days after repeated administration, Fig. 3(B) demonstrates the discrimination of two GTHCs from seven NGTHCs and 10 NGTNHCs with PC1 by PCA, where GTHCs exhibit PC1 below −3.729 (DEN-low, PC1: −3.729, PC2: −1.776), and NGTHCs and NGTNHCs exhibit PC1 above −0.084 (PHE-low, PC1: −0.084, PC2: −1.015) (Appendix C.4). Three intermediate GTHC/NGTHCs (MCT: □, MP: △, and TAA: 〇), MCT-middle (PC1: −4.873, PC2: −1.08), MP-high (PC1: −7.093, PC2: 3.554), TAA-middle (PC1: −4.502, PC2: −1.021), and TAA-high (PC1: −6.931, PC2: 2.398) are in the GTHCs area. MCT-low (PC1: −2.402, PC2: −1.693), MP-middle (PC1: −1.322, PC2: 0.371), and TAA-low (PC1: −1.718, PC2: −0.155) are in the intermediate area between GTHCs and NGTHCs plus NGTNHCs. Only MP-low (PC1: 0.565, PC2: −0.792) is in the NGTHCs and NGTNHCs area. The results of an analysis with 12 genotoxic marker genes [Fig. 3(B)] are similar to those with four genotoxic marker genes [Fig. 2(B)]

At 24 h after a single administration, Appendix C.1 presents the gene expression profile (log2), Appendix C.2 presents the first principal component (PC1) and the second principal component (PC2) analyzed by PCA, and Fig. 3(A) presents the result of PCA of five typical GTHCs, seven typical NGTHCs, 11 NGTNHCs, and three intermediate GTHC/NGTHCs, analyzed with 12 genotoxic marker genes in a two-dimensional graph. The results show a clear separation of five typical GTHCs from seven typical NGTHCs and 11 NGTNHCs by PCA with PC1, where GTHCs exhibit PC1 below –0.381 (DEN-low, PC1: −0.381, PC2: −0.822), and NGTHCs and NGTHCs exhibit PC1 above −0.13 (NGTHC, FEN-high, PC1: −0.13, PC2: 3.184) (Appendix C.2). Speaking of the three intermediate GTHC/NGTHCs, MP-high (PC1: −0.941, PC2: −0.472) and TAA-high (PC1: −3.04, PC2: −0.869) are in the GTHCs area. MCT-high (PC1: −0.109, PC2: −0.353) and TAA-middle (PC1: −0.024, PC2: −1.236) are in the intermediate area. MCT-low (PCA: 1.688, PC2: −0.149), MCT-middle (PC1: 1.149, PC2: −0.489), MP-low (PC1: 1.702, PC2: −0.059), MP-middle (PC1:1.324, PC2: −0.618), and TAA-low (PC1: 1.205, PC2: −0.671) are in the NGTHCs and NGTNHCs area. The analysis with12 genotoxic marker genes are similar to that with four genotoxic marker genes [Fig. 2(A)].

At 29-day after repeated administrations in the previous paper [23], we presented an analysis of 19 chemicals, two typical GTHCs, seven typical NGTHCs, and 10 optional NGTNHCs except for three intermediate GTHC/NGTHCs. Therefore, we only present the result of 19 chemicals plus three intermediate GTHC/NGTHCs, 63 data points, analyzed with 12 previously presented genes in the present paper.

At 29 days, Appendix C.3 presents the gene expression profile (log2), and Appendix C.4 presents the first principal component (PC1) and the second principal component (PC2) analyzed by PCA. Figure 3(B) presents the results of PCA of two typical GTHCs (AAF-low, AAF-middle, AAF-high, DEN-low, and DEN-middle), seven typical NGTHCs with three doses, optional 10 NGTNHCs (AA, ASP, CAF, CPA, CPP, DIA, PBZ, THE, TOL with three doses, and IND-low and IND-middle), and three intermediate GTHC/NGTHCs (MCT-low, MCT-middle, MP-low, MP-middle, MP-high, TAA-low, TAA-middle, and TAA-high), 22 chemicals, 63 points, in a two-dimensional graph. Figure 3(B) demonstrates the discrimination of two GTHCs from seven NGTHCs and 10 NGTNHCs with PC1 by PCA, where GTHCs exhibit PC1 below −3.73 (DEN-low, PC1: −3.729, PC2: −1.776), and NGTHCs and NGTNHCs exhibit PC1 above −0.084 (PHE-low, PC1: −0.084, PC2: −1.015) (Appendix C.4). The distinction between GTHCs and NGTHCs by 12 genes [Fig. 3(B)] and by four genes [Fig. 2(B)] are similar. Speaking of the three intermediate GTHC/NGTHCs, MCT-middle (PC1: −4.873, PC2: −1.08), MP-high (PC1: −7.09, PC2: 3.55), TAA-middle (PC1: −4.50, PC2: −1.02), and TAA-high (PC1: −6.93, PC2: 2.40) are in the GTHCs area. MCT-low (PC1: −2.40, PC2: −1.693), MP-middle (PC1: −1.322, PC2: 0.371), and TAA-low (PC1: −1.72, PC2: −0.155) are in the intermediate area between GTHCs and NGTHCs plus NGTNHCs. Only MP-low (PC1: 0.565, PC2: −0.792) is in the NGTHCs and NGTNHCs area. The results of an analysis with 12 genotoxic marker genes [Fig. 3(B)] are similar to those with four genotoxic marker genes [Fig. 2(B)].

These findings strongly support our recommendation that future rat liver in vivo toxicogenomics tests should prioritize these four genotoxic marker genes, as they have proven to be highly effective in discriminating between different types of hepatocarcinogens.

## Discussion

At the beginning of the twenty-first century, we expected that toxicogenomics approaches would clarify toxic modes of action of chemical compounds, including carcinogens, in a biological system. Although hundreds of studies were published [33, 79–81], practical in vivo short-term screening test methods for carcinogens using toxicogenomics (gene expression profiles) have yet to be established. About 90% of human carcinogens are genotoxic carcinogens [82]; genotoxic carcinogens are still a significant threat to human health. We have been working to develop an in vivo short-term genotoxic carcinogen screening method using gene expression profiles based on the toxic modes of action of chemical compounds and using data analysis by PCA. It would be desirable to distinguish genotoxic carcinogens from non-genotoxic carcinogens, and non-genotoxic noncarcinogens by gene expression profiles and PCA. For this purpose, it is necessary to identify marker genes that distinguish between genotoxic carcinogens, non-genotoxic carcinogens, and non-genotoxic noncarcinogens.

We used PCA for our statistical analysis. PCA is an unsupervised learning algorithm. PCA is not widely used in toxicogenomics but is commonly used in other biological fields, such as cancer analysis [83, 84]. Ringnér wrote that “PCA is often incorporated into genome-wide expression studies.” He explained that “samples can then be plotted, making it possible to visually assess similarities and differences between samples and determine whether samples can be grouped” [85]. PCA is advantageous because the results are clearly understood visually in a two-dimensional or three-dimensional figure with numerical values without bioinformatics knowledge. Previously, we successfully applied PCA to human lung cancer cell lines to discriminate four histopathological subtypes (adenocarcinoma, squamous cell carcinoma, large-cell carcinoma, and small-cell carcinoma) [86, 87]. We also successfully applied PCA to toxicogenomics to discriminate GTHCs from NGTHCs in mice [14], rat models [15, 30–32], and Open TG-GATEs [29].

The present gene data set (Appendix A.1, A.2, A.3, A4; B.1, B.2, B.3, and B.4) may be functional in predicting the genotoxicity of hepato-carcinogenicity of new chemicals; we have added our data to for this purpose. Users can add their data [gene expression profile (log2)] in addition to one of our existing data (Appendix A.1, A.3, B.1, and B.3) one by one or in small numbers and conduct PCA (https://www.r-project.org/). For example, comparing Appendix A.2 and Appendix B.2, even with the addition of the 9 data points, for MCT24hL, MCT24hM, MCT24hH, MP24hL, MP24hM, MP24hH and TAA24hL, TAA24hM, TAA24hH for intermediate GTHC/NGTHCs, the PC1 for AAF24hL in GTHCs does not change significantly from −3.283 (Appendix A.2) to −3.462 (Appendix B.2).

Users can also attempt to calculate PC1 for their test compound using the following formula. The first principal component (Y1) is given by the linear combination of the variable X1, X2, —, Xp.

Y1 = a11X1 + a12X2 + … + a1pXp where a1p is the eigenvector, which can be calculated with the PCA program in R, and Xp is the canonicalized logarithmic (log2)-transformed gene ratio (exp/cont), [(x-μ)/σ].

x is the logarithmic log2 of exp/cont, μ is the mean and σ is the standard deviation (https://strata.uga.edu/software/pdf/pcaTutorial. pdf).

When we calculate all data of 24 h and 29 days by R, a11, –-, a1p of PC1 are a(Bax): −0.501, a(Btg2): −0.500, a(Ccng1): −0.516, a(Cdkn1a): −0.483. Users can calculate their PC1 (Y1) by introducing their xp into the following equation:

$$\mathrm Y1\;=\;(-0.501)\;\mathrm x\;\lbrack(\mathrm{xBax}-0.320)/\;0.0.790\rbrack\;+\;(-0.500)\;\mathrm x\;\lbrack(\mathrm{xBtg}2-0.262)/0.946\rbrack\;+\;(-0.516)\;\mathrm x\;\lbrack(\mathrm{xCcng}1-0.562)/1.1166\rbrack\;+\;(-0.483)\;\mathrm x\;\lbrack(\mathrm{xCdkn}1\mathrm a-0.402)/1.352\rbrack$$

In this equation, users can judge a possibility of GTHC by the cut-off value of −0.507 (median value between the maximum value of GTHC, DEN24hL,—1.168 and the minimum value of NGTHC and NGTNHC, IND24hH, −0.778) obtained in our experiment set, assuming that the difference of μ and σ in [(x-μ)/σ] will be small when total number of samples is greater. We also present the table to calculate the more accurate PC1. Users can add their data (Gene expression profiles (exp/cont) to excel data, then they will get their PC1 value together with a re-calculated cut-off value for GTHC, by integrating new data.

Similarly, the PC2 (Y2) value can be calculated by the following equation:

$$\mathrm Y2\;=\;(0.516\;\mathrm x\;\lbrack(\mathrm{xBax}-0.320)/\;0.790\rbrack\;+\;0.202\;\mathrm x\;\lbrack(\mathrm{xBtg}2-0.262)/0.946\rbrack\;+\;0.079\;\mathrm x\;\lbrack(\mathrm{xCcng}1-0.562)/1.166\rbrack\;+\;(-0.828)\;\mathrm x\;\lbrack(\mathrm{xCdkn}1\mathrm a-0.402)/1.352\rbrack$$

Recently, we published a paper to evaluate rat GTHC and NGTHC via selected gene expression patterns in the liver, as determined by NGS-targeted mRNA sequencing (RNA-Seq) and PCA [30]. We analyzed two typical GTHC (DEN and 3,3’-dimethylbenzidine·2HCl), a typical NGTHC [di-(2-ethylhexyl)phthalate], and 1,4-dioxan (DO), which has long been unclear whether it is a GTHC. The results suggested that PCA discriminated between two GTHCs and NGTHC and that DO resulted in an intermediate gene expression profile different from typical GTHC and NGTHC. In the "Discussion" of the paper, we showed that existing data from TG-GATEs helped evaluate new RNA-Seq data by PCA.

We also performed FFPE RNA-Seq to compare a typical GTHC, 2-acetylaminofluorene (AAF), to genotoxicity equivocal p-cresidine (CRE). CRE is used as a synthetic chemical intermediate, and this compound is classified as an IARC 2B carcinogen and is mutagenic in the *Salmonella* test, which is non-genotoxic to rat livers as assessed by single-strand DNA damage analysis. PCA resulted in CRE as an NGTHC in our experiment. Our results suggest that FFPE RNA-Seq and PCA are useful for evaluating rat GTHCs and NGTHCs [31].

As described previously [29], in connection with restrictions on animal use, “OECD Guidelines for the Testing of Chemicals, [Repeated Dose 28-Day Oral Toxicity Study on Rodents (OECD TG, 2008, 407)] [Test No. 407: Repeated Dose 28-Day Oral Toxicity Study in Rodents | READ online (oecd-ilibrary.org)] is still valid for testing chemical toxicity. This assay determines the general toxicity of chemicals in rodents after 28 days of oral dosing (e.g., effects on the liver, kidney, heart, and lungs). Despite restrictions on animal testing, this test will continue to be applied. We can use the animal organs from the test collaboratively and the samples, reducing the number of experimental animals used. Using FFPE samples is also available for RNA-Seq and spatial transcriptomic [88] and helps to reduce the number of experimental animals used.

Recently, Gi et al. reported ten genotoxic marker genes (*Aen*, *Cdln1a*, *Phlda3*, *Nudt5*, *Mybl1*, *Glrx3*, *Atp6v1f, Mok*, *Cyria*, *Sugct*) from the Open TG-GATEs (with five genotoxic hepatocarcinogens) [89]. *Aen*, *Cdln1a*, *and Phlda3* are common with our previous 12 genotoxic marker genes [14, 29]. We first selected the 12 marker genes with eight different mouse hepatocarcinogens and then evaluated them with rat Open TG-GSTEs. Since the hepatocarcinogens that initially chose the marker genes are different, different marker genes were likely chosen.

Next-generation risk assessment of chemical substances is expected to utilize mechanistic information without animal testing. In this regard, toxicogenomics has proven to be a valuable tool for elucidating the mechanisms underlying the adverse effects of toxic substances: 3D liver microtissue, primary human hepatocytes (PHH) [90], human liver tissue: cancer-derived cell lines (HepaRG) [91], and others are under investigation. The current in vivo short-term test will be instrumental in selecting the genes to be analyzed.

Toxicogenomics technology has progressed from DNA microarray through qPCR to RNA-Seq. DNA microarray and qPCR compare relative fluorescence intensities, while RNA-Seq compares digital nucleotide numbers, which is more reliable. The following emerging technology is the spatial transcriptome (spatial biology) [92]. The technique can count the number of mRNA expressions for each cell in a pathological section. Incorporating this method would provide reliable data because this method can count the number of mRNA expressions for each cell type in the organ. Whether for RNA-Seq or spatial biology, the small number of target genes is an advantage. The four genes in this paper will be helpful for future research. Although the current research on these genes focus only in liver, it is worth extending their application to other organs because the genotoxic mechanism involving them can be common in all organs.

## Conclusions

The present results unequivocally demonstrate the performance of four genotoxic marker genes (*Bax*, *Btg2*, *Ccng1*, and *Cdkn1a*) and PCA in discriminating GTHCs from NGTHCs and NGTNHCs in Open TG-GATEs. These findings strongly support our recommendation that future rat liver in vivo toxicogenomics tests prioritize these four genotoxic marker genes, as they have proven to be highly effective in discriminating between different types of hepatocarcinogens.

## Supplementary Information

Supplementary Material 1.

Supplementary Material 2.

Supplementary Material 3.

## Data Availability

Not applicable.

## Chemicals

AA: Allyl alcohol
AAF: 2-Acetylamidofluorene
AFB: Aflatoxin B1
ɚ-NFL: Alpha-naphthoflavone
ASP: Aspirin
BAL: Balasalazide
BIS: Bisphenol A
CAF: Caffeine
CHL: Chloroform
CLO: Clofibrate
COR: Cortisone
CPA: Chlorpheniramine
CPP: Chlorpropamide
COR: Cortisone
CRE: P-Cresidine
DEHP: Di(2-ethylhexyl)phthalate
DEN: Diethylnitrosamine
DEX: Dexamethasone
DIA: Diazepam
DMF: *N*,*N*-Dimethylformamide
DMN: Dimethylnitrosamine
ETH: Ethanol
FEN: Fenofibrate
FIN: Finasteride
GEN: Gemfibrozil
IFA: Ifosfamide
MCT: Monocrotaline
MP: Methapyrilene
2NF: 2-Nitrofluorene
NNM: *N*-Nitrosomorpholine
PHE: Phenobarbital
PBZ: Phenylbutazone
RAL: Raloxifene
TAA: Thioacetamide
THE: Theophylline
TOL: Tolbutamide

## Genes

*Aen*: Apoptosis enhancing nuclease
*Atp6vaf*: ATPase H+ transporting V1 subunit F
*Bax*: BCL2 associated X, apoptosis regulator
*Btg2*: BTG anti-proliferation factor 2
*Ccnf*: Cyclin F
*Ccng1*: Cyclin G1
*Cgrrf1*: Cell growth regulator with ring finger domain 1
*Cdkn1a*: Cyclin-dependent kinase inhibitor 1A
*Cyria*: CYFIP related Rac1 interactor A
*Gdf15*: Growth differentiation factor 15
*Glrx3*: Glutaredoxin 3
*Lrp1*: LDL receptor related protein 1
*Mdmb*1: Methyl-CpG binding domain protein 1
*Mgmt*: O-6-methylguanine-DNA methyltransferase
*Mok*: MOK protein kinase
*Mybl1*: MYB proto-oncogene like 1
*Nudt5*: Nudix Hydrolase 5
*Phlda3*: Pleckstrin homology-like domain, family A, member 3
*Plk2*: Polo-like kinase 2
*Sugct*: *Succinyl CoA:glutarate-CoA transferase*
*Tmem47*: Transmembrane protein 47
*Tubb4b*: Tubulin, beta 4B class IVb

## Words

EPA: The U.S. Environmental Protection Agency
GTHC: Genotoxic hepatocarcinogen
ICEM: International Conference on Environmental Mutagen
JEMS/MMS: Japanese Environmental Mutagen and Genome Society/Mammalian Mutagenicity Study Group
NGTHC: Non-genotoxic hepatocarcinogen
NGTNHC: Non-genotoxic non-hepatocarcinogen
OECD: The Organisation for Economic Co-operation and Development
PCA: Principal component analysis
PC1: 1St principal component
PC2: 2Nd principal component
qPCR: Quantitative real-time PCR
RNA-Seq: RNA-sequencing
